# Associations Between Accelerometer‐Assessed Sleep Patterns, Proteomic Signatures, and Hallmarks of Aging in Adulthood

**DOI:** 10.1111/acel.70685

**Published:** 2026-08-26

**Authors:** Ruiyi Liu, Jingsong Luo, Yangchang Zhang, Furong Wang, Jing Xu, Wangnan Cao, Shengzhi Sun

**Affiliations:** ^1^ School of Public Health Capital Medical University Beijing China; ^2^ Beijing Key Laboratory of Environment and Aging Capital Medical University Beijing China; ^3^ Beijing Laboratory of Allergic Diseases Beijing Municipal Education Commission Beijing China; ^4^ Department of Social Medicine and Health Education, School of Public Health Peking University Beijing China; ^5^ Department of Rheumatology and Immunology The First Affiliated Hospital of Chongqing University of Chinese Medicine Chongqing China; ^6^ School of Public Health, Key Laboratory of Environmental Pollution Monitoring and Disease Control, Ministry of Education Guizhou Medical University Guiyang China; ^7^ School of Basic Medical Sciences Capital Medical University Beijing China

**Keywords:** accelerometer‐assessed sleep, aging, hallmarks, proteomics, sleep

## Abstract

The associations between objectively measured sleep patterns, the hallmarks of aging, and their shared proteomic signatures remain poorly understood. In this study, we utilized wrist‐worn accelerometer data, plasma proteomic profiles, and health records from the UK Biobank to examine the associations between six defined sleep patterns and nine established hallmarks of aging. We further identified proteins jointly associated with both sleep patterns and aging hallmarks. Longer total sleep duration, greater deep sleep, and increased rapid eye movement (REM) sleep were associated with lower risk of most aging hallmarks. In contrast, higher wakefulness after sleep onset (WASO) and greater sleep irregularity were associated with higher risk, whereas light sleep showed no significant associations. The number of overlapping proteins varied substantially across sleep‐hallmark associations, ranging from 1 to 558. These proteins were predominantly enriched in immune and inflammatory pathways. Several proteins, including IL1RN, FABP1, GDF15, and LEP, were consistently observed across multiple sleep patterns and aging hallmarks. These findings may help generate hypotheses about shared underlying biological processes between sleep and the aging process.

## Graphical abstract

Objectively measured sleep patterns were associated with multiple hallmarks of aging. Total sleep duration, deep sleep, and REM sleep were generally associated with lower risks , whereas longer WASO and sleep irregularity were associated with higher risks. Integrating objective sleep phenotyping with large‐scale proteomics, we identified shared protein signatures related to immune and metabolic pathways.

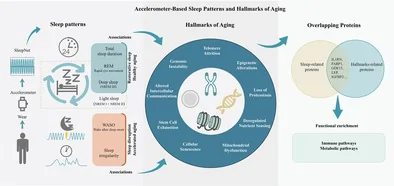

## 1 Introduction

Sleep is increasingly recognized as a fundamental pillar of health (Kohn et al. 2025). Sleep patterns, including duration, quality, and architecture, have been consistently linked to a broad spectrum of health outcomes. Inadequate sleep duration and poor sleep quality are associated with increased risks of cognitive decline, neurodegenerative disorders, immune dysfunction, and cardiovascular diseases (Didikoglu et al. 2020; Garbarino et al. 2021; Högl et al. 2018; Huang 2025; Jackowska and Steptoe 2015; Keil et al. 2023; Mander et al. 2016; Mattis and Sehgal 2016). Additionally, non‐rapid eye movement (NREM) and rapid eye movement (REM) sleep stages represent unique biological processes and relate to disease risk in different ways, suggesting diverse underlying mechanisms (Aurora et al. 2020).

Sleep is closely related to the aging process, influencing immunity and metabolism across systemic, cellular, and genomic scales (Gileles‐Hillel et al. 2016). Previous studies have shown that both short and long sleep durations are associated with accelerated biological age (Wang et al. 2024). Poor subjective sleep quality has also been linked to advanced cellular aging (Cribbet et al. 2014). At the molecular level, sleep disturbances may accelerate biological aging by inducing specific impairments such as telomere attrition and epigenetic changes (Gaspar et al. 2017). However, how objectively measured sleep patterns relate to the cellular and metabolic hallmarks of aging remains poorly characterized, and whether they share proteomic signatures is unclear.

Proteins may serve as key molecular factors associated with both sleep and hallmarks of aging. As biological effectors of genetic and environmental risks, proteins directly reflect physiological processes and homeostatic changes (Deng et al. 2025). For example, obstructive sleep apnea has been associated with elevated levels of 65 proteins involved in complement activation, coagulation, cytokine signaling, and hemostasis (Ambati et al. 2020). Similarly, sleep duration, sleep stages, and rest‐activity rhythm have been linked to protein signatures related to inflammation, apoptosis, glucose homeostasis, and energy metabolism (Ambati et al. 2020; Gonnissen et al. 2012; Svensson et al. 2024; Wei et al. 2025). Despite these findings, evidence on the proteins that are concurrently associated with multiple sleep patterns and aging hallmarks remains scarce. Identifying such proteins could provide insights into the shared molecular correlates of sleep and aging.

In this study, we analyzed accelerometer‐based sleep data from a large UK Biobank cohort, classifying sleep patterns using a validated self‐supervised deep neural network. We examined the associations of these sleep patterns with the hallmarks of aging and focused on proteins concurrently associated with both sleep and aging hallmarks.

## 2 Methods

### 2.1 Study Participants

The UK Biobank is a prospective cohort that recruited more than 500,000 participants aged 40 to 69 years from 22 assessment centers across England, Scotland and Wales between 2006 and 2010 (Palmer 2007). All participants completed a baseline assessment including a touchscreen questionnaire, a computer‐assisted interview covering sociodemographic information, lifestyle, physical measurements, and blood sample collection (Allen et al. 2012; Kianersi et al. 2024). The UK Biobank received ethical approval from the National Information Governance Board for Health and Social Care and the National Health Service North West Centre for Research Ethics Committee (21/NW/0157). Data for this study were obtained from the UK Biobank Resource under Application Number 104745.

Between June 2013 and December 2015, approximately 103,610 participants agreed to participate in an accelerometer sub‐study and completed 7‐day wrist‐worn accelerometry using the Axivity AX3 device on their dominant wrist (Doherty et al. 2017). After quality control, 95,559 participants were retained following the exclusion of those with failed calibration (Data‐Field 90015), non‐wear time exceeding 3 days (Data‐Field 90052), or incomplete data across any 1‐h period of the 24‐h cycle (Data‐Field 90084). We further excluded 2310 participants who had been diagnosed with any of the 83 age‐related diseases (defined below) prior to accelerometer assessment. Disease status and date of first diagnosis were determined using International Classification of Diseases (ICD‐10) codes from hospital inpatient and primary care records. The final analytic sample for the main analyses comprised 93,249 participants (Figure S1).

Proteomic measurements in the UK Biobank were primarily derived from baseline blood samples collected between 2006 and 2010. To identify proteins shared between sleep patterns and aging hallmarks, 9275 individuals with corresponding proteomic data were included (Figure S1).

### 2.2 Accelerometer‐Assessed Sleep Patterns

Sleep patterns were derived using a novel and validated deep learning approach, SleepNet (Yuan et al. 2024). SleepNet employed a multi‐task self‐supervised framework to extract features from 700,000 person‐days of triaxial accelerometry data and was subsequently fine‐tuned using a deep recurrent network to classify sleep patterns against the polysomnography reference standard (Sundararajan et al. 2021; Trevenen et al. 2019; Yuan et al. 2024).

SleepNet was internally validated through subject‐wise five‐fold cross‐validation in the Raine and Newcastle cohorts, followed by external evaluation in the Leicester and Pennsylvania cohorts (Yuan et al. 2024). In external validation against polysomnography, SleepNet achieved an F1 score of 0.66 ± 0.12 for two‐class setting (sleep/wake) and 0.49 ± 0.10 for three‐class setting (wake/REM/NREM) (Yuan et al. 2024), with NREM sleep including stages NREM N1, N2, N3. The differences between SleepNet‐derived estimates and polysomnography were 48.2 min for total sleep duration, −17.1 min for REM sleep duration, and 31.1 min for NREM sleep duration.

Using this validated model, we derived six sleep patterns from the accelerometer data: total sleep duration, REM sleep duration, deep sleep duration (NREM stage N3), light sleep duration (the combined duration of NREM stages N1 and N2), wakefulness after sleep onset (WASO), and sleep irregularity. Sleep irregularity was defined as the 7‐day standard deviation (SD) in sleep duration (Huang et al. 2020; Zheng et al. 2024).

### 2.3 Aging Hallmarks and Age‐Related Diseases

We assessed nine hallmarks of aging using 83 age‐related diseases, based on previous studies (Fraser et al. 2022; Kivimäki et al. 2025; Kuan et al. 2019, 2021). The hallmarks were categorized into four primary and five compensatory processes: genomic instability, telomere attrition, epigenetic alterations, loss of proteostasis, deregulated nutrient sensing, mitochondrial dysfunction, cellular senescence, stem cell exhaustion, and altered intercellular communication. Incident age‐related diseases were identified using the first recorded ICD‐10 codes and their corresponding dates. A complete list of age‐related diseases and the mapping between clinical diagnoses and hallmarks of aging is provided in Tables S1 and S2. Each hallmark was defined by a group of corresponding diseases, and participants diagnosed with at least one of these diseases were considered to exhibit that hallmark. In this study, the nine aging hallmarks served as the primary outcomes, whereas the 83 individual diseases were treated as secondary outcomes.

### 2.4 Proteomic Profiling

Proteomics data were obtained from the UK Biobank Pharma Proteomics Project, which released profiling data for 54,219 baseline plasma samples stored in an automated −80°C sample archive (Sun et al. 2023). A total of 2923 unique proteins were measured using the antibody‐based Olink Explore 3072 platform. Stringent quality control procedures were applied to ensure the quality of protein measurements across cardiometabolic, inflammation, neurology, and oncology panels (Sun et al. 2023). To control for batch and plate effects, normalized protein eXpression (NPX) values were generated using the Olink standard pipeline, which includes a two‐step normalization procedure involving within‐batch and across‐batch intensity normalization (Sun et al. 2023). For our analyses, we excluded proteins with > 50% missing values, resulting in a final set of 2920 proteins. Missing values among the retained proteins were imputed using the cohort‐wide mean (Sun et al. 2023; Zhu et al. 2025).

### 2.5 Covariates

Covariates were selected a priori based on their hypothesized roles as potential confounders of the associations between sleep patterns and hallmarks of aging (Vanderweele 2019; Zhang et al. 2025). All covariates were collected at baseline using standardized and structured questionnaires. We included the following variables: assessment centers, age at enrollment (years), sex (male versus female), ethnicity (White, Black, Asian, Mixed and others), educational levels (college or university degree, General Certificate of Education Advanced Level [A levels]/General Certificate of Education Advanced Subsidiary Level [AS levels] or equivalent, Certificate of Secondary Education [CSEs] or equivalent, General Certificate of Education Ordinary Level [O levels]/General Certificate of Secondary Education [GCSEs] or equivalent, National Vocational Qualification [NVQ]/Higher National Diploma [HND]/Higher National Certificate [HNC] or equivalent, and others) (Hamer et al. 2018), employment status (employed, retired, and other), Townsend deprivation index (TDI; categorized into tertiles), average total household income before tax (< £18,000, £18,000–£30,999, £31,000–£51,999, £52,000–£100,000, > £100,000), body mass index (BMI; underweight [< 18.5 kg/m^2^], normal [18.5–24.9 kg/m^2^], overweight [25.0–29.9 kg/m^2^], and obesity [≥ 30.0 kg/m^2^]), living alone (yes versus no), physical activity (< 500 MET‐min/week, 500–999 MET‐min/week, 1000–1499 MET‐min/week, and ≥ 1500 min/week), smoking status (never, current, previous), and alcohol consumption (never, current, previous). Detailed definitions and coding for all covariates are provided in Table S3.

### 2.6 Statistical Analysis

We used Cox proportional hazards regression models to evaluate associations between the six sleep patterns and nine hallmarks of aging. Follow‐up time was used as the time scale, calculated from the date of accelerometer wear until the first diagnosis of the disease of interest, date of death, or October 31, 2022, whichever occurred first. The proportional hazards assumption was evaluated using Schoenfeld residuals tests and visual inspection of time‐based log hazard ratio (HR) plots; no evidence of departures from this assumption was observed (Figures S2–S7).

Two sequential models were used: a minimally adjusted model including age, sex, and ethnicity, and a fully adjusted model additionally including assessment center, education, TDI, employment, household income, BMI, living alone, physical activity, smoking, and alcohol consumption. Results are presented as HRs with corresponding 95% confidence intervals (CIs). To control for multiple testing, the Benjamini‐Hochberg false discovery rate (FDR) procedure was applied across all models.

In secondary analyses, the same modeling strategy was applied to examine associations between sleep patterns and 83 individual age‐related diseases. To examine the exposure‐response relationship between sleep patterns and aging hallmarks, we modeled sleep patterns using a restricted cubic spline with three degrees of freedom.

To identify proteins associated with both sleep patterns and hallmarks of aging, we first standardized NPX values to *z*‐scores prior to all statistical analyses. Proteomic signatures of sleep patterns were derived using multivariable‐adjusted linear regression (Chadeau‐Hyam et al. 2011). We then identified the proteomic signatures of the nine hallmarks of aging using Cox proportional hazards regression models. All models were adjusted for age, sex, and ethnicity. Overlapping proteins were defined as those significantly associated with both sleep patterns and hallmarks of aging, with significance determined at FDR‐adjusted *p* values < 0.05 in each respective analysis.

We further performed an exploratory analysis to characterize the biological functions of the overlapping proteins. Gene Ontology (GO) enrichment analyses were performed for genes corresponding to the identified overlapping proteins. Enrichment *p* values were adjusted for multiple testing using the FDR method, and redundant GO terms were reduced using a semantic similarity‐based clustering approach with a similarity cutoff of 0.7 (Yu et al. 2012).

To provide a clear overview of our multi‐layered analytical pipeline, a comprehensive roadmap of the entire statistical strategy is presented in Figure S8.

### 2.7 Subgroup Analysis

To evaluate whether the associations between sleep patterns and aging hallmarks varied by sex and age, we conducted subgroup analyses stratified by sex (male and female) and age groups (≤ 50 years, 51–60 years, ≥ 61 years). Heterogeneity across strata was assessed using *p* values for interaction.

### 2.8 Sensitivity Analysis

We performed several sensitivity analyses to assess the robustness of our results. First, to evaluate the potential influence of mental health and other chronic health conditions, we further adjusted the primary Cox models for chronic pain, major depressive disorders, anxiety disorders, and substance‐related disorders (diagnostic codes provided in Table S3). Second, because clinically diagnosed sleep disorders may directly influence accelerometer‐assessed sleep patterns, we repeated the main analyses after excluding participants with any sleep disorder diagnosis recorded before accelerometer assessment. Third, to reduce concerns about reverse causality, we repeated the analyses after excluding individuals who developed the outcome of interest within 180 days or within 2 years of accelerometer assessment. Fourth, to account for the temporal misalignment between proteomic measurements (collected at baseline) and sleep assessment (conducted approximately 5 years later), we additionally adjusted the primary models of the time interval between blood sampling and accelerometry. Fifth, to assess the impact of missing proteomic data, we applied multiple imputation by chained equations with predictive mean matching and repeated the proteomic analyses on the imputed datasets.

All analyses were conducted using R statistical software (version 4.6.0). The “survival” package version 3.2–7 was used for Cox proportional hazards regression, and the “stats” package version 4.6.0 was used for linear regression.

## 3 Results

A total of 93,249 participants (mean age 56.2 ± 7.8 years; 56.3% female) with complete accelerometer data from the UK Biobank were included in the main analyses (Table 1). The study population comprised 58,169 (62.4%) individuals (54.3% female) diagnosed with one or more of the nine hallmarks. The prevalence of specific hallmarks ranged from 35.9% for epigenetic alterations to 54.4% for cellular senescence (Table S4).

**TABLE 1:** Demographic characteristics of the 93,249 UK Biobank participants with and without hallmarks of aging.

Characteristics	Total (*n* = 93,249)	Participants without hallmarks (*n* = 35,080)	Participants with hallmarks (*n* = 58,169)
Age, mean ± SD	56.2 ± 7.8	52.8 ± 7.5	58.2 ± 7.3
Sex			
Female	52,537 (56.3%)	20,977 (59.8%)	31,560 (54.3%)
Male	40,712 (43.7%)	14,103 (40.2%)	26,609 (45.7%)
Ethnicity			
White	90,012 (96.5%)	33,810 (96.4%)	56,202 (96.6%)
Black	787 (0.8%)	284 (0.8%)	503 (0.9%)
Asian	1,060 (1.1%)	416 (1.2%)	644 (1.1%)
Mixed	506 (0.5%)	225 (0.6%)	281 (0.5%)
Others	884 (0.9%)	345 (1.0%)	539 (0.9%)
Education levels^a^			
College or university degree	40,044 (42.9%)	17,231 (49.1%)	22,813 (39.2%)
A levels/AS levels	12,218 (13.1%)	5,076 (14.5%)	7,142 (12.3%)
CSEs or equivalent	3,708 (4.0%)	1,487 (4.2%)	2,221 (3.8%)
O levels/GCEs	18,943 (20.3%)	6,550 (18.7%)	12,393 (21.3%)
NVQ or HND or HNC	4,983 (5.3%)	1,495 (4.3%)	3,488 (6.0%)
Others	12,971 (13.9%)	3,141 (9.0%)	9,830 (16.9%)
Missing	382 (0.4%)	100 (0.3%)	282 (0.5%)
Employment			
Employed	57,418 (61.6%)	26,093 (74.4%)	31,325 (53.9%)
Retired	29,209 (31.3%)	6,499 (18.5%)	22,710 (39.0%)
Others	6,622 (7.1%)	2,488 (7.1%)	4,134 (7.1%)
Average total household income before tax (pound)			
Less than 18,000	12,338 (13.2%)	3,155 (9.0%)	9,183 (15.8%)
18,000 to 30,999	20,292 (21.8%)	6,249 (17.8%)	14,043 (24.1%)
31,000 to 51,999	23,981 (25.7%)	9,455 (27.0%)	14,526 (25.0%)
52,000 to 100,000	20,965 (22.5%)	9,864 (28.1%)	11,101 (19.1%)
Greater than 100,000	6,044 (6.5%)	3,278 (9.3%)	2766 (4.8%)
Missing	9,629 (10.3%)	3,079 (8.8%)	6,550 (11.3%)
Townsend Deprivation index^b^			
T1	31,016 (33.3%)	11,784 (33.6%)	19,232 (33.1%)
T2	31,070 (33.3%)	11,611 (33.1%)	19,459 (33.5%)
T3	31,057 (33.3%)	11,642 (33.2%)	19,415 (33.4%)
Missing	106 (0.1%)	43 (0.1%)	63 (0.1%)
Body Mass Index (kg/m^2^)			
Underweight (< 18.5)	526 (0.6%)	242 (0.7%)	284 (0.5%)
Normal (18.5–24.9)	36,115 (38.7%)	16,275 (46.4%)	19,840 (34.1%)
Overweight (25.0–29.9)	38,289 (41.1%)	13,656 (38.9%)	24,633 (42.3%)
Obesity (≥ 30)	18,115 (19.4%)	4,862 (13.9%)	13,253 (22.8%)
Missing	204 (0.2%)	45 (0.1%)	159 (0.3%)
Summed MET for all activity (MET‐min/week)			
< 500	10,904 (11.7%)	3,972 (11.3%)	6,932 (11.9%)
500 to 1000	11,916 (12.8%)	4,540 (12.9%)	7,376 (12.7%)
1000 to 1500	10,686 (11.5%)	4,283 (12.2%)	6403 (11.0%)
≥ 1500	43,618 (46.8%)	16,865 (48.1%)	26,753 (46.0%)
Missing	16,125 (17.3%)	5,420 (15.5%)	10,705 (18.4%)
Smoking			
Never	53,131 (57.0%)	21,912 (62.5%)	31,219 (53.7%)
Current	6,455 (6.9%)	2,263 (6.5%)	4,192 (7.2%)
Previous	33,420 (35.8%)	10,829 (30.9%)	22,591 (38.8%)
Missing	243 (0.3%)	76 (0.2%)	167 (0.3%)
Alcohol			
Never	2,707 (2.9%)	880 (2.5%)	1,827 (3.1%)
Current	87,907 (94.3%)	33,427 (95.3%)	54,480 (93.7%)
Previous	2,548 (2.7%)	744 (2.1%)	1,804 (3.1%)
Missing	87 (0.1%)	29 (0.1%)	58 (0.1%)
Living a lone			
No	77,587 (83.2)	29,643 (84.5%)	47,944 (82.4%)
Yes	15,477 (16.6)	5,370 (15.3%)	10,107 (17.4%)
Missing	185 (0.2)	67 (0.2%)	118 (0.2%)

Abbreviation: SD, standard deviation.

^a^ A levels/AS levels = General Certificate of Education Advanced Level [A levels]/General Certificate of Education Advanced Subsidiary Level [AS levels] or equivalent; CSEs or equivalent = Certificate of Secondary Education [CSEs] or equivalent; O levels/GCEs = General Certificate of Education Ordinary Level [O levels]/General Certificate of Secondary Education [GCSEs] or equivalent; NVQ or HND or HNC = National Vocational Qualification [NVQ]/Higher National Diploma [HND]/Higher National Certificate [HNC] or equivalent.

^b^ Participants were categorized into tertiles of Townsend Deprivation Index (T1 = least deprived, T3 = most deprived).

**FIGURE 1:**
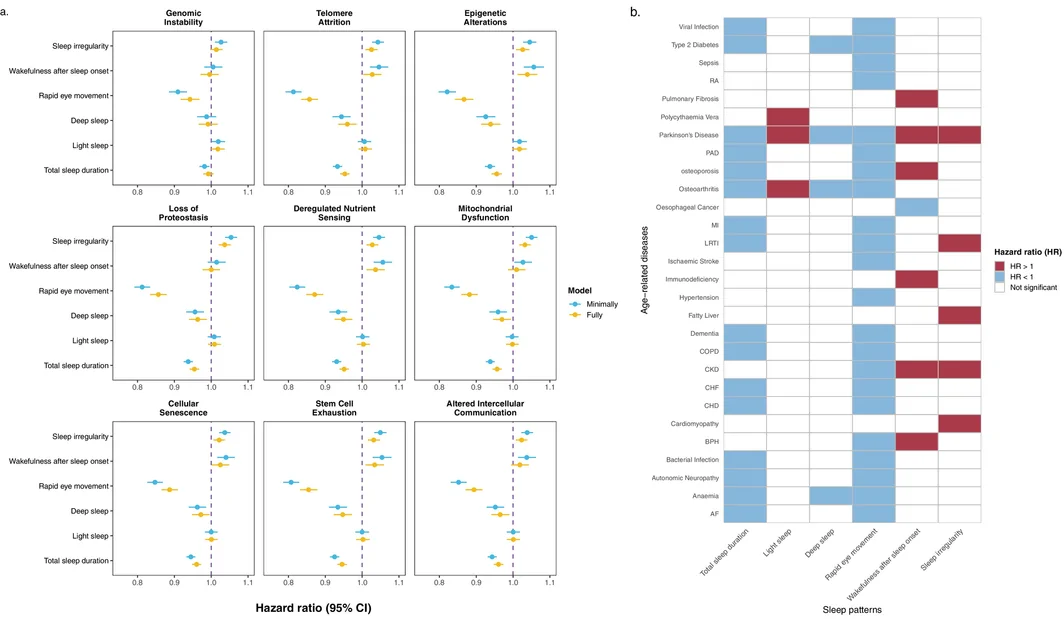
Associations between sleep patterns, hallmarks of aging, and age‐related diseases. (a) Forest plots showing hazard ratios (HRs) from Cox models for the associations between sleep patterns and hallmarks of aging, with false discovery rate (FDR) correction applied to the corresponding *p* values. The minimally adjusted model included age, sex, and ethnicity, whereas the fully adjusted model additionally accounted for assessment center, education level, Townsend deprivation index (TDI), employment status, household income, BMI, living alone and lifestyle factors. (b) Heatmap showing HRs from Cox proportional hazards models for the associations between sleep patterns and age‐related diseases after FDR correction. Red and blue cells indicate associations with HRs > 1 and < 1, respectively, that were statistically significant after FDR correction (FDR‐adjusted *p* value < 0.05); white cells indicate associations that were not statistically significant. All models were adjusted for age, sex, ethnicity, assessment center, education level, TDI, employment status, household income, BMI, living alone and lifestyle factors.

In fully adjusted main analyses, total sleep duration, deep sleep duration, and REM sleep duration were associated with reduced risks of most hallmarks of aging after FDR correction (Figure 1a). For example, each additional hour of total sleep duration was associated with a lower risk for stem cell exhaustion (HR 0.95, 95% CI: 0.93 to 0.96) and deregulated nutrient sensing (HR 0.95, 95% CI: 0.94 to 0.96). Similarly, longer deep sleep duration was associated with lower risks for most hallmarks, particularly epigenetic alterations (HR 0.94, 95% CI: 0.91 to 0.97). Each additional hour of REM sleep was also associated with lower risks across all hallmarks, with the strongest effect observed for stem cell exhaustion (HR 0.85, 95% CI: 0.83 to 0.88). In contrast, greater WASO and higher sleep irregularity were associated with increased risks of several hallmarks, with the largest effects observed for deregulated nutrient sensing (HR 1.04, 95% CI: 1.01 to 1.06) and loss of proteostasis (HR 1.04, 95% CI: 1.02 to 1.05), respectively. No significant associations were observed between light sleep duration and any of the hallmarks (Table S5).

Restricted cubic spline analyses indicated exposure‐response relationships between several sleep patterns and multiple hallmarks of aging (Figures S9–S17). Notably, light sleep, deep sleep, REM sleep, and sleep irregularity generally showed U‐shaped relationships with most hallmarks of aging (all *p* for nonlinearity < 0.001), whereas the remaining associations were approximately linear.

In secondary analyses examining 83 individual age‐related diseases, total sleep duration, deep sleep, and REM sleep were primarily associated with decreased risks, whereas light sleep, WASO, and sleep irregularity were primarily associated with increased risks. After FDR correction, the number of associated diseases varied across sleep patterns: total sleep duration was associated with 16 diseases, light sleep with 3, deep sleep with 4, REM with 22, WASO with 7, and sleep irregularity with 5 (Figure 1b). These associations mainly involved metabolic, circulatory, respiratory, infectious, and neurodegenerative diseases. For example, each additional hour of total sleep duration was associated with a 24% lower risk of Parkinson's disease (HR 0.76, 95% CI: 0.69 to 0.84) (Table S6).

**FIGURE 2:**
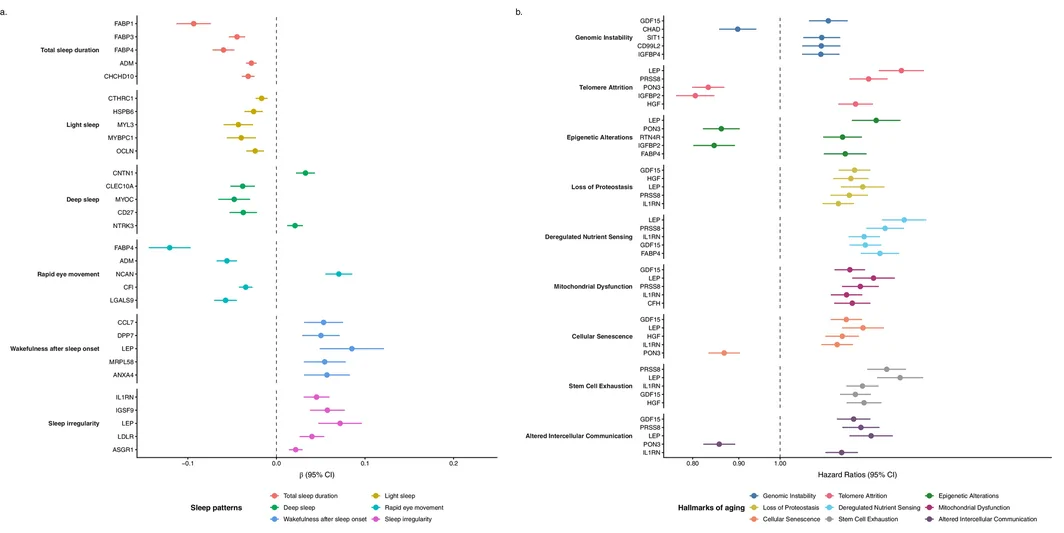
Associations of plasma proteins with accelerometer‐assessed sleep patterns and hallmarks of aging. (a) Forest plot showing the top five proteins associated with each of the six accelerometer‐assessed sleep patterns, ranked by false discovery rate (FDR)‐adjusted *p* values among significant associations (FDR‐adjusted *p* values < 0.05). Points and horizontal lines represent *β* coefficients and 95% confidence intervals, respectively. (b) Forest plot showing the top five proteins associated with each of the nine hallmarks of aging, ranked by FDR‐adjusted *p* values among significant associations (FDR‐adjusted *p* values < 0.05). Points and horizontal lines represent hazard ratios (HRs) and 95% confidence intervals, respectively. All models were adjusted for age, sex, and ethnicity.

Among the 9275 participants with available proteomic data, multivariable linear regression models identified between 32 and 1307 proteins significantly associated with each sleep pattern after FDR correction (Table S7). The largest number of associations was observed for total sleep duration (*n* = 1307), followed by REM sleep (*n* = 1262), sleep irregularity (*n* = 408), WASO (*n* = 403), deep sleep (*n* = 139), and light sleep (*n* = 32). Several proteins, including FGF21, LEP, FABP1, FABP4, and DEFB4A_DEFB4B, were associated with multiple sleep patterns (Table S7). FDR‐adjusted *p* value rankings identified LEP and FABP4 as recurrent top‐five proteins across sleep patterns (Figure 2a).

For the hallmarks of aging, the number of proteins that remained significant after FDR correction varied widely across hallmarks, ranging from 27 to 730. Deregulated nutrient sensing yielded the highest number of proteins (*n* = 730) and genomic instability showed the fewest (*n* = 27) (Table S8). Several proteins, including LEP, PRSS8, FABP4, GDF15, and CDHR2, were associated with multiple hallmarks (Table S8). Based on FDR‐adjusted *p* values, LEP and GDF15 ranked among the top five proteins for seven and eight hallmarks, respectively (Figure 2b).

**FIGURE 3:**
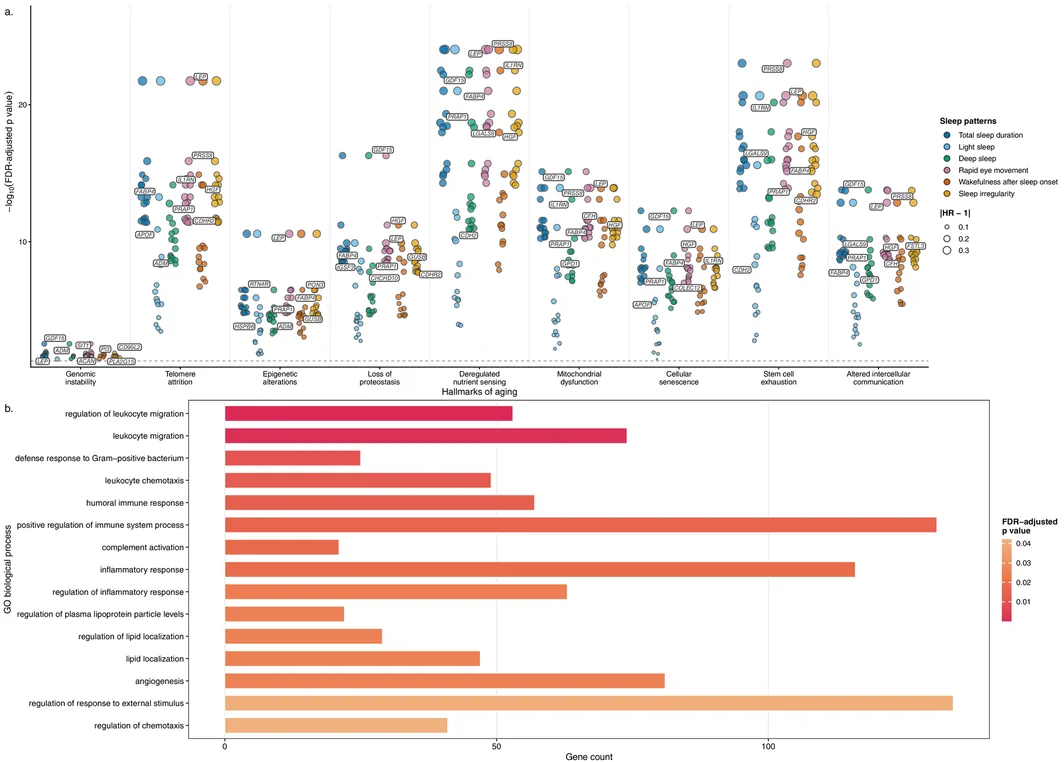
Overlapping proteins associated with both sleep patterns and hallmarks of aging, with exploratory pathway enrichment analysis. (a) Bubble plots showing proteins significantly associated with both sleep patterns and hallmarks of aging (false discovery rate [FDR]‐adjusted *p* value < 0.05 in both analyses). For each sleep pattern‐hallmark combination, the 15 overlapping proteins with the smallest FDR‐adjusted *p* values for the protein‐hallmark associations are shown. The *x*‐axis represents hazard ratios (HRs), and the *y*‐axis represents −log_10_ (FDR‐adjusted *p* value). Point size represents the absolute difference between HR and 1, and colors indicate sleep patterns. For each combination, the top three proteins with the largest |HR−1| values are labeled. (b) Exploratory pathway enrichment analysis of the pooled set of unique overlapping proteins. Pathways remaining significant after FDR correction (FDR‐adjusted *p* value < 0.05) are shown.

Overlapping proteins, defined as those significantly associated with both a sleep pattern and a hallmark, were identified across multiple sleep pattern‐hallmark combinations (Figure 3a). The number of overlapping proteins varied substantially, ranging from 1 (light sleep and genomic instability; LEP) to 558 (total sleep duration and deregulated nutrient sensing) (Table S9). Ranking by FDR‐adjusted *p* values for each sleep‐hallmark combination, several proteins, including LEP, PRSS8, FABP4, GDF15, IL1RN, and PRAP1, were observed across multiple combinations (Figure 3a). Overlapping proteins were predominantly enriched in immune‐ and inflammation‐related pathways, including inflammatory response, complement activation, leukocyte migration, and regulation of chemotaxis (Figure 3b). Exploratory enrichment analyses additionally identified pathways involved in lipid regulation and angiogenesis.

While most associations were consistent between male and female, the associations of total sleep duration and sleep irregularity with epigenetic alterations were more pronounced among female participants (FDR‐adjusted *p* for interaction = 0.03 for both; Table S10). The associations of deep sleep with epigenetic alterations and sleep irregularity with cellular senescence also differed across age groups (FDR‐adjusted *p* for interaction = 0.03 for both; Table S10). No evidence of effect modification by sex or age was found for the remaining associations.

Sensitivity analyses confirmed the robustness of our findings. Results remained consistent after additional adjustment for mental health and chronic conditions (Table S11); excluding participants with sleep disorders (Table S12); excluding early events (Tables S13 and S14); adjusting for the blood sampling–accelerometry time interval (Table S15); and multiple imputation of missing proteomic data (Tables S16 and S17).

## 4 Discussion

In this large prospective study of 93,249 UK Biobank participants with objectively measured accelerometer‐assessed sleep data, we found that longer total sleep duration, deep sleep, and REM sleep were associated with lower risks across most aging hallmarks and age‐related diseases, whereas WASO and sleep irregularity were associated with increased risks. Light sleep, by contrast, showed no significant associations with any hallmarks in the primary analyses. We identified numerous overlapping proteins significantly associated with both sleep patterns and hallmarks of aging, with predominant enrichment in immune and inflammatory pathways.

Our findings extend prior work on individual sleep patterns and aging biomarkers by systematically evaluating six objectively measured sleep patterns across nine established hallmarks of aging. Total sleep duration, deep sleep, and REM sleep were consistently associated with lower risks of most hallmarks of aging, including telomere attrition, epigenetic alterations, cellular senescence, and stem cell exhaustion. These results align with and extend previous evidence connecting sleep to specific molecular aging signatures, including DNA methylation clocks, telomere length, and epigenetic alterations (Carroll et al. 2017; Fostitsch et al. 2025; Kusters et al. 2024; Lahtinen et al. 2019; Morawska et al. 2021; Primiano et al. 2021; Yang et al. 2023). Importantly, the associations for REM sleep and total sleep duration were generally consistent across sex and age groups, suggesting that these sleep patterns may be broadly relevant to biological aging rather than being limited to specific subpopulations.

Conversely, we found that higher WASO and sleep irregularity were associated with higher risks of several hallmarks of aging. These results are consistent with previous studies in middle‐aged and older adults linking greater WASO to telomere shortening (Lehodey et al. 2026) and irregular sleep to accelerated epigenetic aging (Davinelli et al. 2026).

We found little evidence of associations between light sleep and the hallmarks of aging. Light sleep in our study combined accelerometer‐ assessed N1 and N2 sleep. N1 is the transitional phase between wakefulness and sleep, whereas N2 is characterized by the presence of sleep spindles and K‐complexes (Parhizkar and Holtzman 2025). Their associations with aging biomarkers may therefore differ; longitudinal changes in N2 sleep proportion, but not N1 sleep proportion, were weakly associated with telomere shortening (Tempaku et al. 2025). Pooling these estimated stages may have obscured stage‐specific associations. Nevertheless, light sleep showed no significant associations with any hallmark in the primary analyses, in contrast to REM and deep sleep. These findings highlight the value of considering sleep‐stage composition alongside total sleep duration when examining age‐related health (Mander et al. 2017).

Restricted cubic spline analyses showed nonlinear exposure‐response relationships for several sleep‐stage measures and sleep irregularity across multiple hallmarks of aging. For the sleep‐stage measures, these patterns may reflect the interdependence of individual stages within overall sleep architecture. For sleep irregularity, risk generally increased from low to moderate levels and then plateaued, broadly consistent with previous evidence for nonlinear relationships between sleep irregularity and cardiometabolic outcomes (Huang 2025; Kianersi et al. 2024). Given the limited evidence, particularly for sleep stage durations, these findings should be interpreted cautiously and warrant further investigation.

A key strength of our approach lies in the operationalization of aging hallmarks through clinically diagnosed diseases. This top‐down, phenotype‐driven framework, following Kivimäki et al. (2025), serves as a pragmatic proxy for the molecular and cellular hallmarks originally proposed by López‐Otín et al. (2013). Although clinically heterogeneous diseases may cluster within the same hallmark category, this grouping is supported by prior evidence suggesting that conditions assigned to a given hallmark may share convergent biological pathways (Fraser et al. 2022). This biologically informed framework provides an approach to integrating cellular aging concepts with population‐level epidemiological analyses, enabling systematic investigation of how modifiable exposures such as sleep relate to the multifaceted aging process.

Beyond the composite hallmark outcomes, our disease‐level analyses revealed that longer total sleep duration, deep sleep and REM sleep were associated with reduced risks across a broad spectrum of age‐related conditions, including cardiovascular, metabolic, neurological, and endocrine diseases. These findings are consistent with established evidence linking sleep to cardiometabolic health (Chaput et al. 2007; Shan et al. 2015), neurodegenerative risk (Sabia et al. 2022), and endocrine regulation (Nôga et al. 2024). Conversely, WASO and sleep irregularity emerged as risk factors for multiple disease categories, highlighting the potential relevance of sleep continuity and regularity to age‐related health.

However, the effect sizes of these associations were generally modest. Although our findings suggest that sleep patterns are broadly associated with clinical age‐related diseases, the clinical relevance of these associations remains uncertain. Future clinical and intervention studies are needed to determine whether improving sleep patterns can lead to clinically meaningful reductions in the risk of age‐related diseases.

A major contribution of this study is the systematic identification of proteins that are concurrently associated with both sleep patterns and aging hallmarks. We identified several top‐ranked sleep‐associated proteins, including LEP, FABP4, and FGF21, that are established regulators of adiposity, insulin signaling, and metabolic stress responses (Clemmons 2016; Emanuelli et al. 2014; Taheri et al. 2004; Terra et al. 2011). Their associations with total sleep duration, REM sleep, and sleep irregularity suggest that alterations in sleep architecture and regularity may be accompanied by disruptions in metabolic homeostasis and energy regulation (Huang 2025; Morrison et al. 2022). In parallel, we observed prominent associations for inflammatory and immune‐related proteins, including IL6, IL1RN, DEFB4A_DEFB4B, and LGALS9, consistent with well‐documented associations between sleep disturbances and chronic low‐grade inflammation (Garbarino et al. 2021; Irwin 2019).

The overlap between sleep‐ and hallmark‐associated proteins, together with the enrichment results, identifies immune and inflammatory signaling as a potential shared molecular signature of sleep patterns and age‐related processes. Chronic sleep deficiency may sustain a pro‐inflammatory state that, over time, contributes to the cellular and tissue damage characteristic of aging hallmarks. Among the overlapping proteins, LEP is particularly noteworthy given its dual roles in circadian regulation and sleep architecture (Westerterp‐Plantenga 2016) and in metabolic and inflammatory signaling, positioning it as a compelling molecular candidate at the nexus of sleep and aging.

Several limitations should be considered. First, the observational design precludes definitive causal inference, and although sensitivity analyses adjusted for selected mental health and chronic conditions and excluded early events, we could not account for detailed medication use or comprehensive comorbidity burden in the primary models, leaving residual confounding possible. Second, sleep was assessed over a single 7‐day period, which may not capture habitual patterns, long‐term fluctuations, or seasonal variability, potentially introducing non‐differential measurement error and attenuating observed associations. Third, accelerometer data were collected approximately 5 years after baseline blood sampling, creating a temporal gap that prevents interpretation of the overlapping proteins as definitive mediators. Furthermore, despite FDR correction, several proteomic associations may reflect statistical rather than biological significance given the large number of proteins examined. Fourth, sleep stages were estimated using the validated SleepNet algorithm rather than polysomnography, the gold standard. Although the algorithm shows moderate agreement, misclassification is likely and may attenuate stage‐specific estimates. Accordingly, these measures should be viewed as algorithm‐derived operational categories rather than direct polysomnography‐defined sleep architecture, and stage‐specific findings should be interpreted with caution. Fifth, aging hallmarks were proxied by ICD‐coded diseases rather than direct biological biomarkers, which may incompletely capture underlying molecular aging processes. Sixth, the accelerometer sub‐cohort may not be fully representative of the general population, as participants who wore devices had more favorable socioeconomic and health profiles than the broader UK Biobank sample (Zisou et al. 2026), and the predominantly White European composition limits generalizability to more diverse populations. Seventh, despite rigorous quality control including normalization and batch correction of proteomic data, residual technical variability cannot be entirely excluded.

## 5 Conclusion

In this large prospective study of 93,249 UK Biobank participants with objective accelerometer‐assessed sleep data, we observed that favorable sleep patterns, specifically longer total sleep duration, deep sleep, and REM sleep, were associated with lower risks of most aging hallmarks and age‐related diseases, whereas WASO and sleep irregularity were associated with increased risks. By integrating objective sleep phenotyping with large‐scale proteomics, we identified shared immune and metabolic protein signatures. These findings identify proteins associated with both sleep and biological aging, which may help generate hypotheses about shared biological processes and highlight the importance of sleep architecture in the context of healthy aging.

## Author Contributions

**Ruiyi Liu:** conceptualization, writing – original draft, formal analysis, data curation, investigation. **Jingsong Luo:** writing – original draft, review and editing. **Yangchang Zhang:** writing – review and editing. **Furong Wang:** writing – review and editing. **Jing Xu:** writing – review and editing. **Shengzhi Sun:** conceptualization, data curation, methodology, Supervision, writing – review and editing. **Wangnan Cao:** conceptualization, data curation, methodology, Supervision, writing – review and editing.

## Funding

This study was supported by the National Natural Science Foundation of China (82373535).

## Conflicts of Interest

The authors declare no conflicts of interest.

## Supporting information


**Figure S1:** Flowchart of study population selection.
**Figure S2:** Schoenfeld residuals plots: Total sleep duration and hallmarks of aging.
**Figure S3:** Schoenfeld residuals plots: Deep sleep and hallmarks of aging.
**Figure S4:** Schoenfeld residuals plots: Light sleep and hallmarks of aging.
**Figure S5:** Schoenfeld residuals plots: Rapid eye movement and hallmarks of aging.
**Figure S6:** Schoenfeld residuals plots: Wakefulness after sleep onset and hallmarks of aging.
**Figure S7:** Schoenfeld residuals plots: Sleep irregularity and hallmarks of aging.
**Figure S8:** Overview of analytical strategy and multiple testing correction.
**Figure S9:** Exposure‐response relationships between sleep patterns and genomic instability.
**Figure S10:** Exposure‐response relationships between sleep patterns and telomere attrition.
**Figure S11:** Exposure‐response relationships between sleep patterns and epigenetic alterations.
**Figure S12:** Exposure‐response relationships between sleep patterns and loss of proteostasis.
**Figure S13:** Exposure‐response relationships between sleep patterns and deregulated nutrient sensing.
**Figure S14:** Exposure‐response relationships between sleep patterns and mitochondrial dysfunction.
**Figure S15:** Exposure‐response relationships between sleep patterns and cellular senescence.
**Figure S16:** Exposure‐response relationships between sleep patterns and stem cell exhaustion.
**Figure S17:** Exposure‐response relationships between sleep patterns and altered intercellular communication.

**Table S1:** Top 30 ranked age‐related diseases per aging hallmark.
**Table S2:** Mapping of clinical diagnoses to hallmarks of aging.
**Table S3:** UK Biobank covariates.
**Table S4:** Prevalence of hallmarks of aging, stratified by age.
**Table S5:** Associations between sleep patterns and 9 hallmarks of aging.
**Table S6:** Associations between sleep patterns and 83 age‐related diseases.
**Table S7:** Associations between sleep patterns and proteomic signatures.
**Table S8:** Associations between hallmarks of aging and proteomic signatures.
**Table S9:** Numbers of overlapping proteins in sleep patterns and hallmarks of aging.
**Table S10:** Multiplicative interactions of sleep patterns with sex and age in relation to hallmarks of aging.
**Table S11:** Associations between sleep patterns and hallmarks of aging after adjusted for chronic pain, major depressive disorders, anxiety disorders, and several substance‐related disorders.
**Table S12:** Associations between sleep patterns and hallmarks of aging after excluding individuals with sleep disorders before accelerometer assessment.
**Table S13:** Associations between sleep patterns and hallmarks of aging among individuals who developed the condition after 180 days.
**Table S14:** Associations between sleep patterns and hallmarks of aging among individuals who developed the conditions after 2 years.
**Table S15:** Associations between sleep patterns and hallmarks of aging after adjusted time interval between baseline blood sampling and accelerometry assessment.
**Table S16:** Associations between sleep patterns and proteomic signatures using multiple imputation by chained equations.
**Table S17:** Associations between hallmarks of aging and proteomic signatures using multiple imputation by chained equations.

## Data Availability

The data that support the findings of this study are available from UK Biobank. Restrictions apply to the availability of these data, which were used under license for this study. The data may be accessed by eligible researchers through an approved application to UK Biobank at https://www.ukbiobank.ac.uk/use‐our‐data/apply‐for‐access/.
