# Supplementary material for: Associations Between Accelerometer‐Assessed Sleep Patterns, Proteomic Signatures, and Hallmarks of Aging in Adulthood
Source: Aging Cell. 2026 Aug 26;25(9):e70685. doi: 10.1111/acel.70685 (PMC13518371; doi:10.1111/acel.70685)
Supplement: Supplementary file 1 [file ACEL-25-e70685-s002.docx]

**Supplementary Figures**

**Associations between accelerometer-assessed sleep patterns, proteomic signatures, and hallmarks of aging in the UK Biobank**

**Figure S1.** Flowchart of study population selection

**Figure S2.** Schoenfeld residuals plots: Total sleep duration and hallmarks of aging

**Figure S3.** Schoenfeld residuals plots: Deep sleep and hallmarks of aging

**Figure S4**. Schoenfeld residuals plots: Light sleep and hallmarks of aging

**Figure S5.** Schoenfeld residuals plots: Rapid eye movement and hallmarks of aging

**Figure S6.** Schoenfeld residuals plots: Wakefulness after sleep onset and hallmarks of aging

**Figure S7.** Schoenfeld residuals plots: Sleep irregularity and hallmarks of aging

**Figure S8.** Overview of analytical strategy and multiple testing correction

**Figure S9.** Exposure-response relationships between sleep patterns and genomic instability

**Figure S10.** Exposure-response relationships between sleep patterns and telomere attrition

**Figure S11.** Exposure-response relationships between sleep patterns and epigenetic alterations

**Figure S12.** Exposure-response relationships between sleep patterns and loss of proteostasis

**Figure S13**. Exposure-response relationships between sleep patterns and deregulated nutrient sensing

**Figure S14.** Exposure-response relationships between sleep patterns and mitochondrial dysfunction

**Figure S15.** Exposure-response relationships between sleep patterns and cellular senescence

**Figure S16.** Exposure-response relationships between sleep patterns and stem cell exhaustion

**Figure S17.** Exposure-response relationships between sleep patterns and altered intercellular communication

**Figure S1.** Flowchart of study population selection

**Figure S2.** Schoenfeld residuals plots: Total sleep duration and hallmarks of aging

**Figure S3.** Schoenfeld residuals plots: Deep sleep and hallmarks of aging

**Figure S4**. Schoenfeld residuals plots: Light sleep and hallmarks of aging

**Figure S5.** Schoenfeld residuals plots: Rapid eye movement and hallmarks of aging

**Figure S6.** Schoenfeld residuals test: Wakefulness after sleep onset and hallmarks of aging

**Figure S7.** Schoenfeld residuals test: Sleep irregularity and hallmarks of aging

**Figure S8.** Overview of analytical strategy and multiple testing correction

**Figure S9. Exposure-response relationships between sleep patterns and genomic instability.** Restricted cubic spline models were used to examine the association of each sleep pattern with the risk of genomic instability. Solid lines represent hazard ratios, and shaded areas represent 95% confidence intervals. The horizontal dashed line indicates a hazard ratio of 1. Models were adjusted for the same covariates as those included in the main analyses.

**Figure S10. Exposure-response relationships between sleep patterns and telomere attrition.** Restricted cubic spline models were used to examine the association of each sleep pattern with the risk of telomere attrition. Solid lines represent hazard ratios, and shaded areas represent 95% confidence intervals. The horizontal dashed line indicates a hazard ratio of 1. Models were adjusted for the same covariates as those included in the main analyses.

**Figure S11. Exposure-response relationships between sleep patterns and epigenetic alterations.** Restricted cubic spline models were used to examine the association of each sleep pattern with the risk of epigenetic alterations. Solid lines represent hazard ratios, and shaded areas represent 95% confidence intervals. The horizontal dashed line indicates a hazard ratio of 1. Models were adjusted for the same covariates as those included in the main analyses.

**Figure S12. Exposure-response relationships between sleep patterns and loss of proteostasis.** Restricted cubic spline models were used to examine the association of each sleep pattern with the risk of loss of proteostasis. Solid lines represent hazard ratios, and shaded areas represent 95% confidence intervals. The horizontal dashed line indicates a hazard ratio of 1. Models were adjusted for the same covariates as those included in the main analyses.

**Figure S13. Exposure-response relationships between sleep patterns and deregulated nutrient sensing.** Restricted cubic spline models were used to examine the association of each sleep pattern with the risk of deregulated nutrient sensing. Solid lines represent hazard ratios, and shaded areas represent 95% confidence intervals. The horizontal dashed line indicates a hazard ratio of 1. Models were adjusted for the same covariates as those included in the main analyses.

**Figure S14. Exposure-response relationships between sleep patterns and mitochondrial dysfunction.** Restricted cubic spline models were used to examine the association of each sleep pattern with the risk of mitochondrial dysfunction. Solid lines represent hazard ratios, and shaded areas represent 95% confidence intervals. The horizontal dashed line indicates a hazard ratio of 1. Models were adjusted for the same covariates as those included in the main analyses.

**Figure S15. Exposure-response relationships between sleep patterns and cellular senescence.** Restricted cubic spline models were used to examine the association of each sleep pattern with the risk of cellular senescence. Solid lines represent hazard ratios, and shaded areas represent 95% confidence intervals. The horizontal dashed line indicates a hazard ratio of 1. Models were adjusted for the same covariates as those included in the main analyses.

**Figure S16. Exposure-response relationships between sleep patterns and stem cell exhaustion.** Restricted cubic spline models were used to examine the association of each sleep pattern with the risk of stem cell exhaustion. Solid lines represent hazard ratios, and shaded areas represent 95% confidence intervals. The horizontal dashed line indicates a hazard ratio of 1. Models were adjusted for the same covariates as those included in the main analyses.

**Figure S17. Exposure-response relationships between sleep patterns and altered intercellular communication.** Restricted cubic spline models were used to examine the association of each sleep pattern with the risk of altered intercellular communication. Solid lines represent hazard ratios, and shaded areas represent 95% confidence intervals. The horizontal dashed line indicates a hazard ratio of 1. Models were adjusted for the same covariates as those included in the main analyses.
